# Targeting the sensory feedback within the swallowing network—Reversing artificially induced pharyngolaryngeal hypesthesia by central and peripheral stimulation strategies

**DOI:** 10.1002/hbm.25233

**Published:** 2020-10-17

**Authors:** Paul Muhle, Bendix Labeit, Andreas Wollbrink, Inga Claus, Tobias Warnecke, Carsten H. Wolters, Joachim Gross, Rainer Dziewas, Sonja Suntrup‐Krueger

**Affiliations:** ^1^ Department of Neurology with Institute of Translational Neurology University Hospital Muenster Muenster Germany; ^2^ Institute for Biomagnetism and Biosignalanalysis University Hospital Muenster Muenster Germany

**Keywords:** dysphagia, neuromodulation, pharyngeal electrical stimulation, pharyngeal pneumatic stimulation, swallowing, transcranial direct current stimulation

## Abstract

Pharyngolaryngeal hypesthesia is a major reason for dysphagia in various neurological diseases. Emerging neuromodulation devices have shown potential to foster dysphagia rehabilitation, but the optimal treatment strategy is unknown. Because functional imaging studies are difficult to conduct in severely ill patients, we induced a virtual sensory lesion in healthy volunteers and evaluated the effects of central and peripheral neurostimulation techniques. In a sham‐controlled intervention study with crossover design on 10 participants, we tested the potential of (peripheral) pharyngeal electrical stimulation (PES) and (central) transcranial direct current stimulation (tDCS) to revert the effects of lidocaine‐induced pharyngolaryngeal hypesthesia on central sensorimotor processing. Changes were observed during pharyngeal air‐pulse stimulation and voluntary swallowing applying magnetoencephalography before and after the interventions. PES induced a significant (*p* < .05) increase of activation during swallowing in the bihemispheric sensorimotor network in alpha and low gamma frequency ranges, peaking in the right premotor and left primary sensory area, respectively. With pneumatic stimulation, significant activation increase was found after PES in high gamma peaking in the left premotor area. Significant changes of brain activation after tDCS could neither be detected for pneumatic stimulation nor for swallowing. Due to the peripheral cause of dysphagia in this model, PES was able to revert the detrimental effects of reduced sensory input on central processing, whereas tDCS was not. Results may have implications for therapeutic decisions in the clinical context.

## Graphical abstract

Pharyngolaryngeal hypesthesia is a major reason for dysphagia in various neurological diseases. Here, we investigated whether pharyngeal electrical stimulation (PES) or transcranial direct current stimulation can revert the effects of experimentally induced pharyngeal hypesthesia on cortical activation in healthy adults in a crossover sham‐controlled design by means of magnetoencephalography. PES was able to revert the detrimental effects of reduced sensory input on central processing, whereas transcranial direct current stimulation was not.

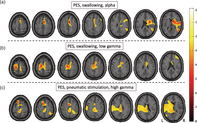

**Abbreviations**
BA: Brodmann area
ERD: event‐related desynchronization
MEG: magnetoencephalography
MTT: maximum tolerated threshold
PES: pharyngeal electrical stimulation
PT: perceptual threshold
SMA: supplementary motor area
tDCS: transcranial direct current stimulation
TMS: transcranial magnetic stimulation

## 1 INTRODUCTION

Apart from a finely tuned oropharyngeal muscular contraction pattern involving more than 25 different muscles, the act of swallowing is highly dependent on unimpaired sensory feedback (Muhle, Suntrup‐Krueger, & Dziewas, 2018). The swallowing network is constantly modulated by the continuous sensory feedback from the oral cavity and the pharynx via cranial nerves V, IX, and X to adapt the motor program to environmental conditions. Thus, characteristics of the bolus, such as volume or viscosity, lead to a modulation of the motion sequence of swallowing. A larger bolus, for example, induces an earlier hyolaryngeal elevation as well as an earlier opening of the upper esophageal sphincter compared to a smaller bolus (Cook et al., 1989; Jacob, Kahrilas, Logemann, Shah, & Ha, 1989). Finally, protective mechanisms, in particular, clearing swallows dealing with pharyngeal residues as well as a reflexive cough, are critically dependent on intact sensory feedback (Aviv et al., 1996; Borders et al., 2019; Onofri, Cola, Berti, da Silva, & Dantas, 2014; Shapira‐Galitz, Shoffel‐Havakuk, Halperin, & Lahav, 2019). In the clinical context, several studies have shown that pharyngeal hypesthesia is associated with complications and bad outcome. In stroke patients, a strong correlation between sensory deficit and dysphagia severity has been described (Marian et al., 2017) putting these patients at an increased risk of pulmonary infections secondary to aspiration (Rohweder, Ellekjar, Salvesen, Naalsund, & Indredavik, 2015). In tracheotomized patients, severely impaired laryngeal sensation is among the key reasons why after successful respiratory weaning removal of the tracheal cannula needs to be postponed (Warnecke et al., 2013). Finally, in the context of critical care medicine, dysphagia after extubation, the most important driver of extubation failure, is also linked to pharyngolaryngeal sensory deficits (Macht, Wimbish, Bodine, & Moss, 2013; Suntrup‐Krueger et al., 2019). The key role of afferent sensory information is also highlighted by new and emerging treatment strategies in this clinical domain. Thus, capsaicin and other pharmaceutical agents enhancing sensory input have been shown to improve swallowing safety in patients with dysphagia (Wirth & Dziewas, 2019). On the same note, pharyngeal electrical stimulation (PES) is supposed to promote rehabilitation of neurogenic dysphagia—at least in part—by restoring peripheral sensory feedback (Suntrup, Marian, et al., 2015; Suntrup‐Krueger et al., 2016). Thus, in a randomized‐controlled trial recruiting tracheostomized stroke patients suffering from severe dysphagia, compared to sham stimulation, PES significantly increased the proportion of patients who were ready for decannulation after study intervention (Dziewas et al., 2018). However, because neurophysiological and functional imaging studies are difficult to conduct in these severely ill neurological patients, the underlying physiological principles involved in the restoration of sensory feedback and related central sensory processing in particular in PES are still only poorly understood. Therefore, in the present study, we made use of a previously developed “virtual lesion model” inducing transient pharyngeal and laryngeal hypesthesia in healthy adults and leading to both impaired swallowing performance and altered central sensorimotor processing (Labeit et al., 2019; Muhle, Claus, et al., 2018; Teismann et al., 2007). To revert the detrimental effects of peripheral hypesthesia on the central processing of sensory input and the swallowing motor program, we adopted PES as a peripheral stimulation tool and compared its effects to that of transcranial direct current stimulation (tDCS) applying magnetoencephalography (MEG). tDCS is a well‐recognized central stimulation strategy that already has shown promising results in treating dysphagia poststroke (Suntrup‐Krueger et al., 2018b). We hypothesized that because of the peripheral cause of dysphagia in this model, PES may show more robust effects than tDCS in this experimental setup.

## 2 MATERIALS AND METHODS

### 2.1 Experimental outline

To investigate whether tDCS and PES can reverse the effects of experimentally induced pharyngeal hypesthesia on the cortical swallowing network, MEG was recorded during two previously established tasks for each neurostimulation method: Isolated effects on the sensory pathway were identified by delivering air‐puffs to the pharyngeal wall (Muhle, Claus, et al., 2018). Effects on entire cortical swallowing processing were assessed during a voluntary swallow task (Suntrup, Teismann, Wollbrink, et al., 2013). Thus, four different conditions were studied in the MEG in subjects with local pharyngeal hypesthesia (see also online supplementary Figure S1):

1. voluntary swallowing at baseline and after (real or sham) tDCS;
2. pharyngeal pneumatic stimulation at baseline and after (real or sham) tDCS;
3. voluntary swallowing at baseline and after (real or sham) PES;
4. pharyngeal pneumatic stimulation at baseline and after (real or sham) PES.

In this sham‐controlled study with a crossover design, there were two separate measurement sessions for each condition: subjects were randomly assigned to receive either sham stimulation or the real intervention on the first session and vice versa in the following session.

### 2.2 Subjects

Ten healthy volunteers were studied (five females, mean age: 29.0 ± 6.8 years). No participant took any medication that affects the central nervous system and subjects had no history of dysphagia, neurologic, ear‐nose‐throat, or psychiatric disorders.

### 2.3 Interventions

#### 2.3.1 Transcranial direct current stimulation

tDCS was delivered by a constant current stimulator (NeuroConn GmbH, Ilmenau, Germany) using a pair of conductive‐rubber electrodes encased in two saline‐soaked sponges, as previously described (Suntrup, Teismann, Wollbrink, et al., 2013). Based on evidence from previous studies (Suntrup, Teismann, Wollbrink, et al., 2013; Suntrup‐Krueger et al., 2018a), the center of the anode (5 × 7 cm) was placed approximately 3.5 cm to the right and 0.5 cm posterior to the vertex with its long axis parallel to the central sulcus to target the right primary sensorimotor cortex for swallowing. The right hemisphere was chosen for stimulation because it is preferentially concerned with the coordination of the pharyngeal phase of swallowing, whereas the left hemisphere is more connected to the oral phase (Suntrup, Teismann, Wollbrink, et al., 2013). We therefore assumed right‐sided stimulation to be optimal to revert experimentally induced pharyngeal hypesthesia and related pharyngeal phase dysphagia in our subjects. The reference electrode (10 × 10 cm) was placed over the left orbit. Anodal tDCS was performed with a current strength of 1.5 mA resulting in current densities of 0.043 mA/cm^2^ (anode) and 0.015 mA/cm^2^ (cathode). These stimulation parameters had previously shown to revert central inhibition (“virtual lesion”) of the human pharyngeal motor cortex induced by transcranial magnetic stimulation (TMS) (Vasant et al., 2014). According to this best available evidence, we chose to apply the same treatment parameters in our peripheral sensory virtual lesion model. In the intervention session, the current was slowly ramped up over 30 s and stimulation was continued for a total of 9 min before decreasing the current again over 30 s. In the sham condition, current flow was ramped up and down for 30 s to evoke a similar tingling sensation to the initiation of real tDCS. Then the electrodes were left in place for a further 9:30 min without current flow. During either stimulation session, subjects sucked on a lemon‐flavored lollipop to activate sensory afferents and to increase the swallowing rate because tDCS is generally said to show the best effects if combined with specific activation of the targeted brain network.

#### 2.3.2 Pharyngeal electrical stimulation

PES was performed as previously described (Suntrup, Teismann, et al., 2015) using a transnasally inserted intraluminal catheter with a 3.2 mm diameter that houses a pair of bipolar platinum ring electrodes 10 mm apart (Gaeltec, Ltd., Dunvegan, Isle of Skye, UK). The catheter was connected to a constant current stimulator (Model DS7A) controlled by a trigger generator (Model DG2A, both Digitimer Limited, Welwyn‐Garden City, Herts, UK). In brief, electrical stimulation was delivered with a pulse duration of 0.2 ms at a frequency of 5 Hz with 280 V, which had previously shown optimal effects in a dose–response‐study (Fraser et al., 2003). To ensure sufficient stimulation intensity, as well as good subject tolerance the perceptual threshold (PT) and the maximum tolerated threshold (MTT) for stimulation intensity was determined individually three times by slowly increasing the current in either the sham and the intervention sessions. The average values of three trials were used for the individual calculation of the optimal stimulation intensity according to the formula PT + 0.75 × (MTT − PT) (Fraser et al., 2003). The PT was also used as a measure to confirm sufficient pharyngeal hypesthesia after topical application of lidocaine (see below). In the intervention sessions, stimulation was delivered at the calculated intensity for a total of 10 min whereas in the sham condition the catheter was left in place for a further 10 min without current flow.

### 2.4 Experimentally induced pharyngeal hypesthesia

**FIGURE 1:**
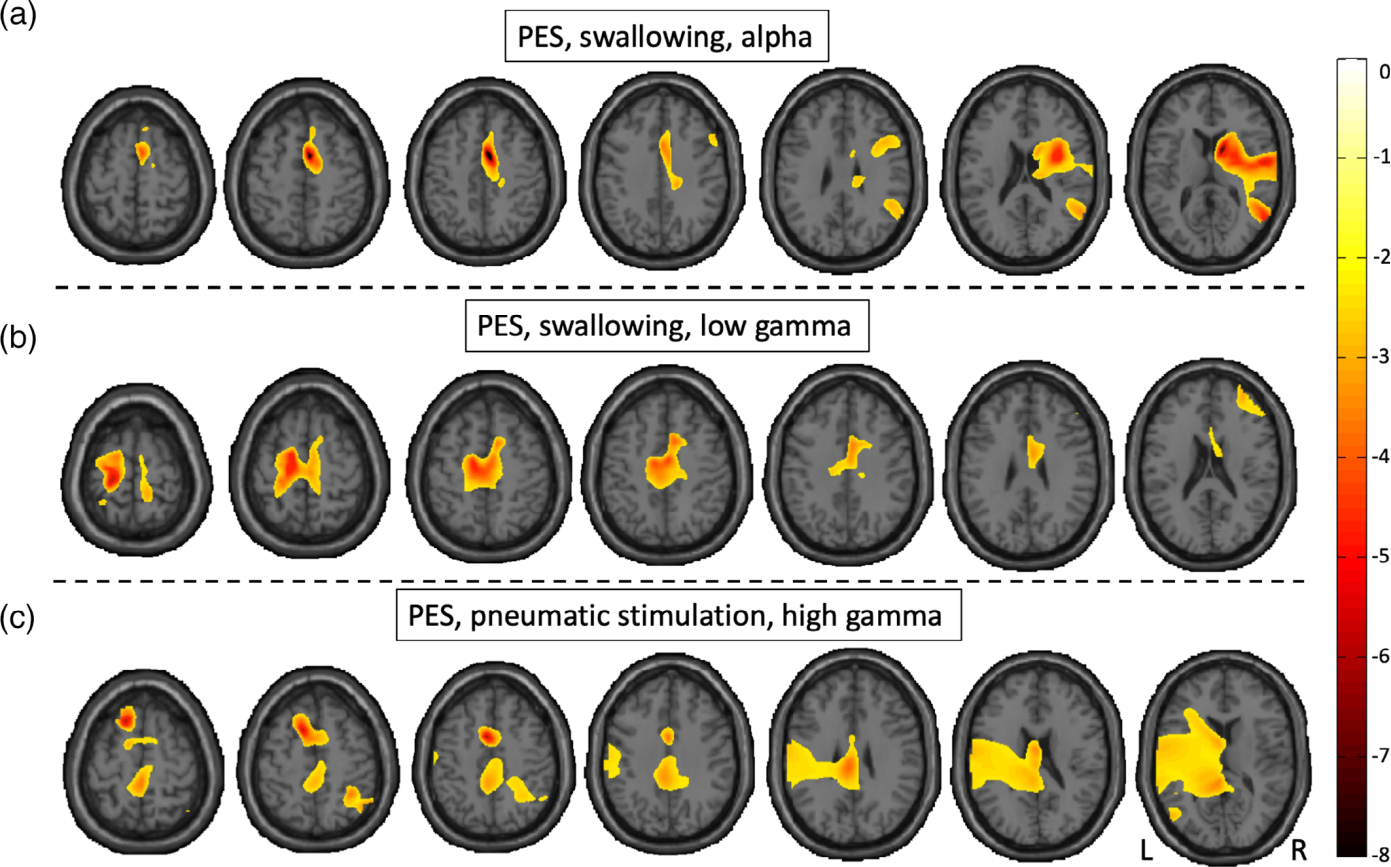
Brain areas with color‐coded, significant increase of event‐related desynchronization (ERD) after real as compared to sham pharyngeal electrical stimulation (PES) during swallowing (a,b) and pneumatic stimulation (c). Negative values denote ERD, *p* < .05, L = left, R = right

To induce a virtual peripheral sensory lesion before the neurostimulation interventions, five sprays of lidocaine (10 mg per spray) were applied to either side of the pharynx in our subjects, leading to topical hypesthesia for at least half an hour (Muhle, Claus, et al., 2018). As described in that preceding study (Muhle, Claus, et al., 2018), this design leaves sufficient time for 10 min of neurostimulation to revert the virtual lesion plus further 5 min to reposition the subject in the MEG chamber after the intervention and 15 min MEG measurement time. To ensure comparability between baseline and postintervention MEG results, the anesthetic was applied 15 min prior to the beginning of MEG recording for both baseline and poststimulation measurements (Figure 1). Moreover, in the PES conditions, baseline PT was determined prior to the first lidocaine application. Sufficient hypesthesia was confirmed by retesting of PT after spraying the full lidocaine dose mentioned above. An increase of 50–80% of baseline PT was targeted as a virtual lesion model of disease‐related pharyngeal hypesthesia. Another testing of PT was performed after the second lidocaine application prior to the PES intervention (Figure 1) to confirm a similar grade of hypesthesia with again 50–80% increase of baseline PT. Repetition of topical anesthesia was necessary for our experimental setup because its effect diminishes over time.

### 2.5 MEG data acquisition and analysis

MEG data were acquired using a whole head 275‐channel SQUID sensor array (Omega 275, CTF systems Inc.) with well‐established recording parameters and experimental tasks (Muhle, Claus, et al., 2018; Suntrup, Teismann, Wollbrink, et al., 2013; Suntrup, Teismann, et al., 2015; Suntrup‐Krueger et al., 2018a; Teismann et al., 2011). The sample frequency was 600 and a 150 Hz low‐pass filter was used. Subjects were seated in an upright position in the MEG chamber. To keep the state of alertness on a constant level, subjects were watching a silent movie in the MEG during all conditions. During the 15 min MEG measurement of the voluntary swallowing task, water was continuously infused in the oral cavity via a plastic tube (4.7 mm in diameter) at a flow rate of 10 ml/min. Participants were asked to swallow volitionally without external cueing. Pneumatic pharyngeal stimulation was delivered in a separate session for 15 min as previously established by our group (Muhle, Claus, et al., 2018; Teismann, Steinstraeter, et al., 2009). In summary, air‐pulses (pressure: 3 bar, pulse duration: 500 ms; frequency: 0.5 Hz, stimulus onset jitter: 600 ms) were delivered to the pharyngeal wall via a baby nasogastric tube (8 French, Nutrisafe2, Vygon, France) inserted through the right nostril which was connected to a compressor outside of the MEG chamber. Auditory masking with “pink noise” via in‐ear headphones was used to prevent stimulus‐related activation of the auditory system when receiving pneumatic stimulation (Muhle, Claus, et al., 2018).

To detect swallowing acts for later event‐related analysis in conditions A and C and for later rejection of pneumatic stimulation trials contaminated by swallowing artifacts in conditions B and D, surface electromyographic (EMG) recording electrodes were placed on the submental muscle groups (Suntrup, Teismann, et al., 2015; Teismann et al., 2007; Teismann et al., 2011) and the EMG signal was coregistered with the MEG. Data were preprocessed and analyzed with our established analysis pipeline that has been applied in several previous MEG studies in this field (Muhle, Claus, et al., 2018; Suntrup, Teismann, Wollbrink, et al., 2013; Suntrup, Teismann, et al., 2015). Prior to further computations, MEG data were filtered within five different frequency bands: theta (4–8 Hz), alpha (8–13 Hz), beta (13–30 Hz), low gamma (30–60 Hz), and high gamma (60–80 Hz) by applying a fourth‐order two‐pass Butterworth filter. Custom‐made scripts based on the open‐source MATLAB (MathWorks Inc.) toolbox FieldTrip (http://www.ru.nl/fcdonders/fieldtrip) were used for subsequent data analysis (Oostenveld, Fries, Maris, & Schoffelen, 2011). Swallowing as well as sensory stimulation are known to evoke an event‐related desynchronization (ERD) in respective cortical regions (Pfurtscheller, 2001; Teismann, Steinstraeter, et al., 2009). Source localization of each individual's cortical ERD changes due to swallowing/pneumatic pharyngeal stimulation was performed separately in all frequency bands for each session and condition applying a linearly constrained minimum variance beamformer and a single shell volume conduction model (Nolte, 2003) generated from the canonical single subject T1‐weighted MRI (SPM8, http://www.fil.ion.ucl.ac.uk/spm) implemented in FieldTrip. For the event‐related analysis of the swallowing task, a time window from −0.4 to 0.6 s in reference to the initiation of main swallowing muscle activation as seen in the EMG was taken as active stage whereas a 1 s time interval after each swallow served as resting stage. In pneumatic stimulation datasets, those trials contaminated by swallows were rejected first. In the remaining data, the active stage was defined as 0–500 ms after the pneumatic stimulus and contrasted against a baseline interval from −500 to 0 ms before the stimulus. Next, volumetric source estimation results from the beamformer were spatially normalized to a template brain (T1, Montreal Neurological Institute, Canada) using SPM8. Grandaverages of normalized source activation maps were computed across all subjects for the separate tasks and conditions in each frequency band.

For the identification of significant cortical activation, changes due to stimulation a two‐by‐two factorial design with factors time (pre vs. post) and intervention (real vs. sham stimulation) was used with applying a cluster‐based permutation test. First, baseline versus postintervention contrasts were generated for each task and condition by subtraction. Resulting difference data structures of real versus sham data were afterwards compared separately for each condition and intervention type applying a cluster‐based nonparametric randomization approach (to control for Type I error with respect to multiple comparisons) with a two‐sided dependent samples t‐statistic built into FieldTrip and taking a *p*‐value <.05 as statistically significant (Maris & Oostenveld, 2007). Descriptive statistics and comparison of subject characteristics, as well as behavioral data, were carried out using SPSS Statistics 25.0 (IBM Corp.).

## 3 RESULTS

### 3.1 Task performance

Data on head movement in the MEG, number of analyzed swallows/pneumatic stimuli per dataset before and after the intervention are presented in Table 1. There were no statistically significant differences in MEG measurement performance between baseline and postintervention measurements, except for the sham session of Condition B. Here, subjects moved their head significantly more in the MEG after the intervention compared to the MEG measurement before sham tDCS (*p* = .029). PT, MTT, and stimulation intensity for PES are presented in Table 2. Intended PT increase after lidocaine application between 50 and 80% of baseline PT values was confirmed. PES thresholds and calculated stimulation intensity did not differ significantly between real and sham stimulation sessions. For tDCS, the skin impedance measured by the tDCS device was slightly higher in the sham conditions compared to real stimulation sessions (Condition A: 7.87 ± 3.31 vs. 5.26 ± 1.69 kΩ, *p* = .015; Condition B: 9.75 ± 3.63 vs. 7.03 ± 1.19 kΩ, *p* = .008). No adverse events occurred during the experiments.

**TABLE 1:** Data on number of analyzed events and head movement during MEG according to condition

	Number of analyzed events (±*SD*)			Head movement (mm ± *SD*)		
	Pre	Post	*p*‐Value	Pre	Post	*p*‐Value
Condition A						
Real	58.80 ± 16.785	65.07 ± 16.197	0.503^a^	0.502 ± 0.378	0.401 ± 0.178	0.733^b^
Sham	68.93 ± 23.906	70.33 ± 22.318	0.086^a^	0.446 ± 0.165	0.472 ± 0.281	0.649^a^
Condition B						
Real	409.60 ± 16.668	405.20 ± 20.369	0.492^a^	0.347 ± 0.177	0.409 ± 0.2164	0.225^a^
Sham	420.53 ± 30.437	423.27 ± 18.691	0.950^b^	0.351 ± 0.222	0.465 ± 0.253	0.029^b^
Condition C						
Real	80.90 ± 26.06	77.70 ± 23.71	0.389^a^	0.365 ± 0.146	0.464 ± 0.399	0.445^b^
Sham	75.20 ± 21.78	71.6 ± 20.13	0.465^a^	0.306 ± 0.172	0.385 ± 0.253	0.070^a^
Condition D						
Real	412.10 ± 25.787	413.8 ± 27.804	0.795^a^	0.446 ± 0.400	0.449 ± 0.226	0.980^a^
Sham	410.30 ± 31.885	402.70 ± 36.606	0.683^b^	0.407 ± 0.331	0.380 ± 0.174	0.762^a^

Abbreviations: MEG, magnetoencephalography; min, minutes; mm, millimeter; *SD*, standard deviation.

^a^ Paired *t* test.

^b^ Wilcoxon rank test.

**TABLE 2:** Technical data on PES interventions

	Condition C			Condition D		
	Sham	Intervention	*p*‐Value	Sham	Intervention	*p*‐Value
PT without hypesthesia ± *SD* (mA)	4.77 ± 0.60	4.70 ± 0.88	.806^a^	4.52 ± 0.31	4.37 ± 0.67	.504^a^
PT with hypesthesia prior to baseline MEG ± *SD* (mA)	8.85 ± 1.24	7.90 ± 1.29	.943^a^	7.72 ± 1.09	7.60 ± 1.68	.765^a^
PT with hypesthesia before PES ± *SD* (mA)	8.42 ± 1.10	8.35 ± 1.22	.912^a^	7.80 ± 1.63	7.42 ± 1.09	.517^a^
MTT with hypesthesia before PES ± *SD* (mA)	15.36 ± 3.71	13.29 ± 2.44	.070^a^	13.95 ± 3.91	12.79 ± 4.07	.066^a^
Stimulation intensity ± *SD* (mA)	13.63 ± 3.02	12.11 ± 1.98	.116^a^	12.41 ± 3.31	11.45 ± 3.09	.054^a^

Abbreviations: mA, milliampere; MEG, magnetoencephalography; MTT, maximum tolerated threshold; PES, pharyngeal electrical stimulation; PT, perceptual threshold; *SD*, standard deviation.

^a^ Paired *t* test.

### 3.2 Cortical activation

#### 3.2.1 Baseline data with experimentally induced pharyngeal hypesthesia

Swallowing after local anesthesia (Conditions A and C) lead to ERD of oscillatory brain activity from theta to low gamma frequency range and was most prominent in the alpha and beta frequency bands. Swallowing‐associated activation was localized bilaterally mainly in the pericentral cortex corresponding to primary and secondary sensorimotor areas, as previously described (Teismann et al., 2007). No task‐related activation changes were found in the high gamma range. During all baseline measurements performing swallowing, strongest activation was found in the beta frequency band at the right Brodmann area (BA) 6 (peak‐values for: Condition A_tDCS_ = −0.269; Condition A_sham_ = −0.246; Condition C_PES_ = −0.194; Condition C_sham_ = −0.244. Negative values indicate ERD). Peak activation in the other frequency ranges was: Condition A_tDCS_: alpha = −0.086 (left BA 6), low gamma = −0.096 (right BA 6); Condition A_sham_: alpha = −0.168 (left BA 6), low gamma = −0.102 (right BA 6); Condition C_PES_: alpha = −0.072 (left BA 1), low gamma = −0.082 (right BA 6); Condition C_sham_: alpha = −0.168 (left BA 6), low gamma = −0.102 (right BA 6).

As intended, the withdrawal of sensory afferent information by topical anesthesia leads to a pronounced reduction of cortical responses to pneumatic pharyngeal stimulation. In the grandaverages of individual source distributions, the remaining ERD centered in beta and adjacent alpha and low gamma frequency ranges. Activation following pneumatic stimulation was localized bilaterally mainly in areas corresponding to primary and secondary somatosensory and motor cortices, the supramarginal gyrus as well as the insula. To a lesser extent, activation was also found in prefrontal areas, as previously described (Muhle, Claus, et al., 2018). Activation intensity was relevantly lower than in the swallowing conditions, with the relatively strongest albeit small activation peaks found as follows: Condition B_tDCS_: alpha −0.029 (left cerebellum); beta −0.031 (left BA 6), low gamma −0.018 (right BA 10); high gamma −0.016 (right BA 9); Condition B_sham_: alpha −0.030 (right BA 1), beta −0.018 (BA 4), low gamma −0.014 (left BA 39); high gamma −0.016 (right BA 6); Condition D_PES_: alpha −0.045 (left BA 22); beta −0.026 (left BA 22); low gamma −0.012 (left BA 1); high gamma −0.021 (right BA 37); Condition D_sham_: alpha −0.051 (left BA 18); beta −0.030 (left BA 37); low gamma −0.066 (right BA 10); high gamma: −0.058 (right BA 10). Negative values indicate ERD. Grandaverages of baseline and postintervention data for swallowing and pneumatic pharyngeal stimulation in the PES conditions are displayed in the online supplementary information (supplementary Figures S2–S5).

#### 3.2.2 Intervention effects

##### Transcranial direct current stimulation

Significant changes of brain activation after real or sham tDCS could neither be detected for swallowing nor for pneumatic stimulation by the cluster‐based permutation statistic in any of the analyzed frequency ranges (smallest cluster *p*‐value >.05). Cortical activation changes due to experimentally induced peripheral pharyngeal hypesthesia could not be reverted by this central stimulation method in our study.

##### Pharyngeal electrical stimulation

In Condition C, a significant increase of ERD was induced by real as compared to sham PES in the alpha frequency range peaking in the right supplementary motor area (BA 6) (peak value: −12.694; *p*_*α*_ = 0.047, Figure 1a) as well as in the low gamma frequency range peaking in the left primary sensory cortex (BA1, Figure 1b) (peak value: −6.309; *p*_lowγ_ = 0.039). Further significant activation increase in alpha was found in the right‐hemispheric pre‐ (BA 4) and postcentral gyrus (BA 1, 2, 3, 43), the (pre‐) frontal gyrus (BA 8–10, 45–47), parietal gyrus (BA 39), supramarginal gyrus (BA 40), anterior cingulate (BA 24, 32), insula (BA13), orbitofrontal cortex (BA 11), temporopolar cortex (BA 38), and parts of the temporal lobe (BA 20–22, 41, 42), as well as the occipital lobe (BA 18, 19). In low gamma, main activation increase was observed more bilaterally but with left‐hemispheric dominance in sensorimotor areas (BA 4,6, 1–3) and supramarginal gyrus (BA 40), bilateral frontal cortex (BA 8–10, 45–47), anterior cingulate (BA 24, 32), and right orbitofrontal cortex (BA11).

In Condition D, a statistically significant PES‐induced increase of ERD as compared to sham stimulation was detected in the low gamma frequency range in the left premotor cortex (BA6, peak value: −8.922; p_lowγ_ = 0.010), bilateral but left‐dominant pre‐ (BA 4) and postcentral gyrus (BA 1, 2, 3, 43), left supramarginal gyrus (BA 40), insula (BA 13), the parietal cortex (BA 39), (pre‐) frontal gyrus (BA 8, 9, 10, 46, 47), orbitofrontal cortex (BA 11), and temporal gyri (BA 20, 21, 22) as well as temporopolar cortex (BA 38) (Figure 1c). A significant activation increase was also observed in the anterior cingulate cortex (BA 24) bilaterally and in the right occipital lobe (BA 18, 19).

No significant effects were found in any other frequency band in Conditions C or D. Moreover, there were no brain regions with significant reduction of ERD due to stimulation.

## 4 DISCUSSION

While previous neurophysiological research has mostly focused on the central processing of motor aspects of deglutition, only recently more attention was drawn to the crucial role of intact sensory afferents for the motor output of swallowing. Despite its clinical significance, the effect of neuromodulatory treatment on specific disturbances of the sensory swallowing network has not yet been investigated, which was a primary goal of this study. We found that PES can—at least partly—revert the negative effects of sensory afferent deprivation on cortical processing of pharyngeal sensory stimuli and swallowing, whereas tDCS was not able to influence the central network alterations resulting from experimentally induced pharyngeal hypesthesia in either of these conditions.

### 4.1 Baseline cortical activation in the virtual lesion model

With various functional imaging modalities, swallowing‐associated activation has among other regions most consistently been found in the pericentral cortex bilaterally, congruent with primary and secondary sensorimotor areas (Michou & Hamdy, 2009; Suntrup, Teismann, Wollbrink, et al., 2013; Suntrup, Teismann, et al., 2015). Pharyngeal sensory stimulation has been shown to evoke a similar but more caudolateral sensorimotor cortex activation pattern (Lowell et al., 2008;Muhle, Claus, et al., 2018; Teismann, Steinstraeter, et al., 2009). Findings emphasize a close functional connection between both tasks. Especially the fact that purely sensory stimulation activates both sensory and motor components of the swallowing system (Lowell et al., 2008; Lowell, Reynolds, Chen, Horwitz, & Ludlow, 2012; Muhle, Claus, et al., 2018) stresses the importance of sensory input in cerebral swallowing control. This is further supported by the spatiotemporal characteristics of cortical activation following the application of a single electrical pharyngeal stimulus. Here, the evoked potential first appears in the sensory cortex closely followed by a peak in the motor cortex 5 ms later, indicating an intracortical serial network (Gow, Hobson, Furlong, & Hamdy, 2004).

The peripheral sensory lesion model used in the present study was previously established by our group for swallowing (Teismann et al., 2007) and pharyngeal pneumatic stimulation (Muhle, Claus, et al., 2018; Teismann, Steinstraeter, et al., 2009). Thus, a baseline condition without topical anesthesia was not recorded here. In those preceding studies, we could demonstrate that oropharyngeal hypesthesia leads to a strong decrease of not only sensory but also motor cortical activation during pneumatic pharyngeal stimulation and swallowing in healthy adults, even resulting in worse performance in a water swallow test. As expected and intended, the baseline activation patterns observed in the present study are in line with these former results.

### 4.2 Localization, lateralization and frequency range of intervention effects

The brain areas in which increased activation was found after PES have been described as being activated during swallowing (Furlong et al., 2004; Hamdy, Rothwell, et al., 1999; Hamdy, Mikulis, et al., 1999; Lowell et al., 2012; Malandraki, Sutton, Perlman, Karampinos, & Conway, 2009; Suntrup, Teismann, et al., 2015; Teismann et al., 2007) as well as following sensory stimulation of the pharynx (Lowell et al., 2008; Lowell et al., 2012; Muhle, Claus, et al., 2018; Rofes, Ortega, Vilardell, Mundet, & Clave, 2017; Teismann, Steinstraeter, et al., 2009) in multiple functional neuroimaging studies. In a former MEG study by our group (Suntrup, Teismann, et al., 2015) PES in healthy subjects without pharyngeal hypesthesia lead to changes of ERD in similar sensorimotor brain areas for swallowing as observed here. Likewise, pneumatic pharyngeal stimulation without hypesthesia evokes an activation pattern similar to what we measured after the PES intervention in the present study (Muhle, Claus, et al., 2018). Taken together, PES seems to foster reactivation of the regular cortical swallowing network, which was temporarily downregulated because of sensory deprivation.

The relevance of these reactivated areas for swallowing function and pharyngeal sensory processing shall be described in short. Apart from the significant role of the primary sensorimotor cortex, the initiation of swallowing movement has been associated with the premotor cortex (BA6) and SMA (Nachev, Kennard, & Husain, 2008). The parietal gyrus plays a role in motor attention and is connected to the insula, the primary sensory cortex, and premotor areas (Babaei et al., 2013; Hamdy, Rothwell, et al., 1999; Lowell et al., 2012). The insula as the primary gustatory cortex is involved in the processing of oropharyngeal stimuli. It is functionally connected with sensory and motor areas, immediately suggesting its role for multisensory and sensorimotor integration (Augustine, 1996; Hamdy, Rothwell, et al., 1999; Rolls, 2005) and assumed to be involved in the coordination of the temporal sequence of oropharyngeal movements (Martin, Goodyear, Gati, & Menon, 2001; Mosier, Liu, Maldjian, Shah, & Modi, 1999). The relevance of the temporal lobe is not clear but lesions in this area are associated with pharyngeal residue as dysphagia symptom (Humbert et al., 2009; Mosier et al., 1999; Suntrup‐Krueger et al., 2017; Wilmskoetter et al., 2019). The cingulate is thought to be involved in planning and initiation of voluntary swallowing (Hamdy, Rothwell, et al., 1999; Lowell et al., 2008). The anterior cingulate cortex is connected to autonomic nuclei in the brainstem and involved in visceromotor control (Aziz et al., 2000). BA 18 and 19 as part of the occipital lobe are considered as “visual association area,” their role in swallowing is not yet entirely clear. However, Lowell et al. demonstrated activation in the occipital lobe as well as the supramarginal gyrus (BA 40) following sensory stimulation of the pharynx (Lowell et al., 2008). Employing fMRI, it was shown that both regions, as well as the inferior parietal lobe and SMA, were connected to the primary sensory cortex, indicating their potential relevance for processing of sensory information (Lowell et al., 2012). Eventually, the (pre‐)frontal cortex is suggested to be involved in attentive aspects of swallowing (Hamdy, Mikulis, et al., 1999). The orbitofrontal cortex as part of the prefrontal area receives projections from the primary and secondary somatosensory cortex and the insula. It represents a secondary taste and olfactory cortex and is activated by pleasant or painful touch, also indicating a role for sensory processing (Hagen, Zald, Thornton, & Pardo, 2002; Rolls, 2004).

With regard to the frequency distribution of the observed changes, a reincrease of ERD in alpha and low gamma was observed for the swallowing‐task and in the low gamma range for pneumatic stimulation. The alpha rhythm is generally related to somatosensory processing and integration, whereas, for example, beta oscillations, which were not influenced significantly by PES, are especially linked to the motor components of a task (Andres et al., 1999; Studer, Koeneke, Blum, & Jancke, 2010). Gamma band activity is said to be related to somatosensory processing in primary and secondary somatosensory areas (Ihara et al., 2003). Within sensory systems, integrative processes likely involve corticocortical synchronization of high‐frequency oscillatory activity (Engel, Fries, & Singer, 2001; Fries, 2009). Gamma oscillations seem to constitute a framework that allows carrying cohesive patterns of neural activity along sensory streams (Fries, 2015). It has been proposed that low gamma activity may support afferent sensory feedback to the sensorimotor cortex during the performance of movement. As an example, perceived stimuli create stronger gamma oscillations than unperceived stimuli of equal stimulus intensity (Gross, Schnitzler, Timmermann, & Ploner, 2007). Taken together, these concepts support our interpretation that increased alpha and gamma activity in the present study may indeed represent the restoration of pharyngeal sensory afferent pathways with PES.

The reoccurrence of ERD following pharyngeal stimulation in the “sensory” alpha range was lateralized predominantly to the right‐hemispheric swallowing network, similar to findings from our previous MEG study on PES (Suntrup, Teismann, et al., 2015). This may well be because the right hemisphere is concerned with the sensorimotor processing of the pharyngeal phase of swallowing (Cuellar et al., 2016; Daniels, Foundas, Iglesia, & Sullivan, 1996; Li et al., 2009; Smithard, O'Neill, Martin, & England, 1997; Suntrup, Kemmling, et al., 2015; Suntrup‐Krueger et al., 2017). For instance, there is evidence that activation of the cortical swallowing network is time‐dependent and shifts from the left hemisphere during the early oral stage of swallowing to the right hemisphere during the later stage of swallowing, that is, the pharyngeal phase (Mihai, Otto, Platz, Eickhoff, & Lotze, 2014; Teismann, Dziewas, Steinstraeter, & Pantev, 2009). Since electrical stimulation was performed in the pharynx and not in the oral cavity, an activation increase mainly in the right‐hemispheric “pharyngeal” swallowing network within the “sensory” alpha range with only secondary bilateral upregulation in low gamma seems plausible.

Contrary to the swallowing‐task, PES‐induced rebound of cortical ERD during pneumatic stimulation was lateralized to the left hemisphere, similar to our former MEG studies in which we had implemented the pharyngeal air‐pulse stimulation setup (Muhle, Claus, et al., 2018; Teismann, Steinstraeter, et al., 2009). As the tube to deliver air‐puffs was always inserted through the right nostril, the stimuli were mainly directed to the right side of the pharyngeal wall. Most afferent fibers from the spinal trigeminal nucleus, where the stimulated glossopharyngeal sensory nerve fibers converge on, cross the midline in the brainstem before ascending to the somatosensory cortex (Yoshida, Tanaka, Hirano, & Nakashima, 2000), which, as a consequence, is predominantly activated in the left hemisphere. PES was able to restore this activation pattern in lateralization and localization.

### 4.3 PES versus tDCS

It needs to be discussed why in the present peripheral sensory lesion model PES had a positive treatment effect whereas tDCS had not. Based on the results of previous clinical studies in stroke patients with dysphagia, we hypothesized PES as a peripheral stimulation method to be effective in patients with isolated peripheral pharyngeal hypesthesia or mixed central and peripheral dysphagia etiology whereas cortical tDCS may be especially helpful in patients with central lesions of the swallowing network.

The treatment effect of PES is—besides neuromodulatory facilitation of afferent pathways—at least partly also mediated via local pharyngeal release of Substance P (Suntrup‐Krueger et al., 2016) from sensory nerve fiber terminals in the pharyngeal mucosa. Substance P is a neuropeptide known to enhance the swallowing reflex (Jin et al., 1994). In a clinical intervention trial on PES treatment to foster removal of the tracheal cannula in severely dysphagic, tracheostomized stroke patients an increased Substance P concentration after PES was indicative of decannulation success due to a relevant improvement of swallowing function (Muhle et al., 2017). In these patients who respond well to PES, dysphagia is of mixed central (the stroke lesion itself) and peripheral etiology due to various sequelae of invasive ICU treatment (Macht et al., 2013). The effectiveness of PES in ICU‐treated stroke patients with combined dysphagia etiology is further backed up by two clinical trials that have shown distinctly higher decannulation rates after repetitive PES compared to sham stimulation (Dziewas et al., 2018; Suntrup, Marian, et al., 2015).

DCS, on the other hand, seems to have less potential in case of peripheral damage to the swallowing system and may be especially effective in case of predominantly central swallowing network affection. The method has been shown to improve swallowing function in moderately affected, non‐tracheotomized dysphagic stroke patients (Suntrup‐Krueger et al., 2018a). In a subgroup of patients from that investigation, MEG was even performed presenting an increase of ERD in swallow‐related contralesional areas after application of anodal tDCS over that intact hemisphere. A virtual central lesion to the pharyngeal motor cortex induced by 1 Hz rTMS mimicking a stroke lesion could be removed by contralateral tDCS (Vasant et al., 2014).

Comprising, there are indications that central stimulation approaches aid dysphagia rehabilitation resulting from a central lesion whereas PES seems to be superior with regard to the treatment of peripheral pharyngeal sensory impairment. In the clinical context, both stimulation approaches have shown promising results in stroke patients, in whom dysphagia usually does not occur as a consequence of a central or peripheral lesion alone but rather results from a combination of the two.

### 4.4 Methodological considerations and limitations

We applied a previously developed virtual lesion model to mimic a neurological impairment in healthy subjects (Muhle, Claus, et al., 2018; Teismann et al., 2007), being aware that pathophysiologic mechanism and underlying recovery processes in patients might be different to our healthy volunteers. Because this study was focused on the comparative evaluation of the neurophysiological effects of both neurostimulation techniques, we did not assess potential changes in swallowing function. Subsequent studies—especially in patients—should correlate neurophysiological changes with behavioral gains. Given the complexity of the study, design with eight appointments for each participant sample size was small but within the common range of MEG studies.

In the absence of proven tDCS stimulation protocols targeting the sensory swallowing network, we applied tDCS parameters that had previously been found effective to enhance activation in the pharyngeal motor cortex. It is known that a deafferented sensory cortex becomes hypo‐active but also hyperexcitable due to homeostatic mechanisms as found in TMS studies (Karabanov et al., 2015). In such a condition, the effects of tDCS could be different, or even opposite to what would have been expected. A hyperexcitable cortex could theoretically respond in an inhibitory way to our excitatory, anodal tDCS, due to activation of homeostatic inhibitory mechanisms with protective meaning (cortical metaplasticity). Thus, a different stimulation protocol and/or target area might have been more effective. Critics of tDCS may take the lack of a neurophysiologic effect as argument that the stimulation is currently too unspecific to modulate such a complex network. Novel approaches such as individualized multichannel‐tDCS may contribute to an increase of effect size in the future (Khan et al., 2019; Wagner, Burger, & Wolters, 2016).

Skin impedance was significantly higher during sham stimulation compared to the actual tDCS. Sham tDCS was short lasting and not intended to have any real stimulation effect. Hence, worse impedance is neglectable in this context. As a higher impedance is causing a more intense tingling sensation, it is probable that participants were even more likely to believe they received the actual treatment. PES requires a stimulation intensity above the PT. As a consequence, the sham condition could be discriminated from real PES despite topical anesthesia from our healthy volunteers. To diminish the risk of a bias subjects were not aware of the purpose of the study intervention. Head movement during pneumatic stimulation in the MEG following sham PES (Condition B) was significantly higher compared to baseline, but it was still within an acceptable range of <5 mm. Apart from that, task performance in the MEG and level of anesthesia was comparable between baseline and postintervention sessions, making it unlikely that observed changes in cortical activation result only from variance in the experimental circumstances.

## 5 CONCLUSION

In our peripheral sensory lesion model of dysphagia, PES as a peripheral stimulation method was able to revert the detrimental effects of reduced sensory input on central swallowing processing, whereas tDCS as a central neuromodulation technique was not. Results may have implications for therapeutic decisions depending on the nature of dysphagia in the clinical context.

## CONFLICTS OF INTEREST

R. D. is member of the advisory board of Phagenesis. All other authors declare no conflicts of interest.

## AUTHOR CONTRIBUTIONS

**Paul Muhle**: Investigation, writing–original draft, visualization. **Bendix Labeit**: Investigation, formal analysis. **Andreas Wollbrink**: Methodology, software. **Inga Claus**: Formal analysis, writing–review and editing. **Tobias Warnecke**: writing–review and editing. **Carsten H. Wolters**: writing–review and editing. **Joachim Gross**: Resources, writing–review and editing. **Rainer Dziewas**: Conceptualization, supervision, writing–review and Editing, project administration. **Sonja Suntrup‐Krueger**: Conceptualization, supervision, methodology, writing–original draft, project administration.

## ETHICS STATEMENT

The study protocol was approved by the local ethics committee. Informed consent was obtained from each participant following the principles of the declaration of Helsinki.

## Supporting information


**Supplementary Figure S1** Experimental setup including conditions A – D; tDCS = transcranial direct current stimulation; PES = pharyngeal electrical stimulation
**Supplementary Figure S2**: Swallowing‐related group mean Event‐Related Desynchronization (ERD) before (upper array) and after real PES (lower array) according to frequency bands alpha, beta, low gamma; N = 10. Negative values denote ERD. L = left; R = right
**Supplementary Figure S3**: Swallowing‐related group mean ERD before (upper array) and after sham‐PES (lower array) according to frequency bands alpha, beta, low gamma; N = 10. Negative values denote ERD. L = left; R = right.
**Supplementary Figure S4**: Group mean ERD related to pharyngeal air‐puff stimulation before (upper array) and after real PES (lower array) according to frequency bands alpha, beta, low gamma, high gamma; N = 10. Negative values denote ERD. L = left; R = right.
**Supplementary Figure S5**: Group mean ERD related to pharyngeal air‐puff stimulation before (upper array) and after sham‐PES (lower array) according to frequency bands alpha, beta, low gamma, high gamma; N = 10. Negative values denote ERD. L = left; R = right

## Data Availability

Data and the code from our custom‐made scripts used for MEG analysis are available upon request to the authors with the need for a formal data sharing agreement due to legal restrictions imposed by the University Hospital Muenster.
