# Supplementary material for: Targeting the sensory feedback within the swallowing network—Reversing artificially induced pharyngolaryngeal hypesthesia by central and peripheral stimulation strategies
Source: Hum Brain Mapp. 2020 Oct 17;42(2):427–38. doi: 10.1002/hbm.25233 (PMC7776007; doi:10.1002/hbm.25233)
Supplement: Supplementary file 1 — Supplementary Figure S1 Experimental setup including conditions A – D; tDCS = transcranial direct current stimulation; PES = pharyngeal electrical stimulation Supplementary Figure S2: Swallowing‐related group mean Event‐Related Desynchronization (ERD) before (upper array) and after real PES (lower array) according to frequency bands alpha, beta, low gamma; N = 10. Negative values denote ERD. L = left; R = right Supplementary Figure S3: Swallowing‐related group mean ERD before (upper array) and after sham‐PES (lower array) according to frequency bands alpha, beta, low gamma; N = 10. Negative values denote ERD. L = left; R = right. Supplementary Figure S4: Group mean ERD related to pharyngeal air‐puff stimulation before (upper array) and after real PES (lower array) according to frequency bands alpha, beta, low gamma, high gamma; N = 10. Negative values denote ERD. L = left; R = right. Supplementary Figure S5: Group mean ERD related to pharyngeal air‐puff stimulation before (upper array) and after sham‐PES (lower array) according to frequency bands alpha, beta, low gamma, high gamma; N = 10. Negative values denote ERD. L = left; R = right [file HBM-42-427-s001.pdf]

Online Supplement

Targeting the sensory feedback within the swallowing network – reversing artificially induced pharyngolaryngeal hypesthesia by central and peripheral stimulation strategies

Supplementary figures:

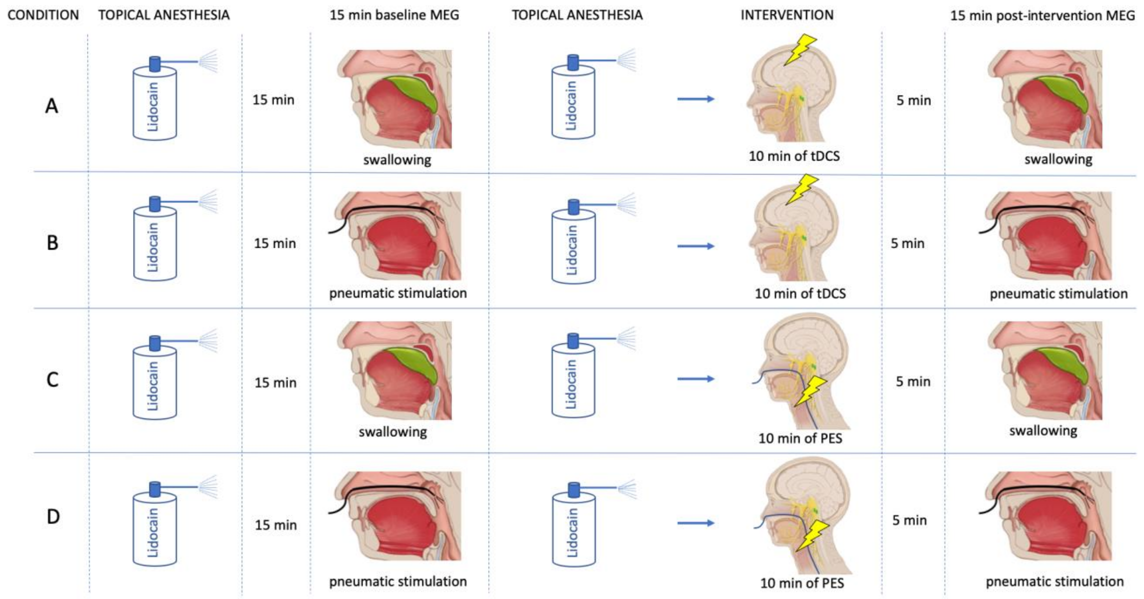

Supplementary figure 1: Experimental setup including conditions A – D; tDCS = transcranial direct current stimulation; PES = pharyngeal electrical stimulation

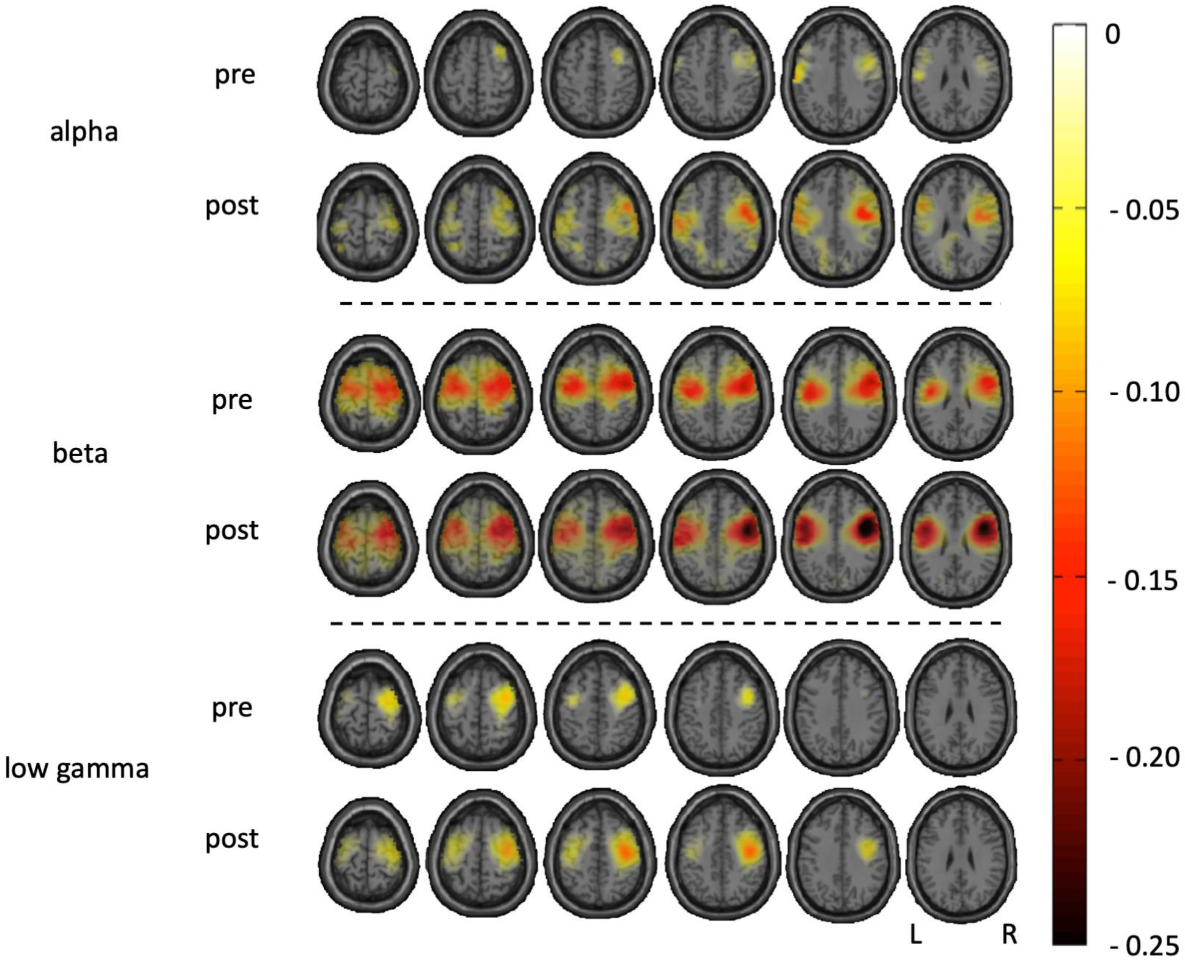

Supplementary figure 2: Swallowing-related group mean Event-Related Desynchronization (ERD) before (upper array) and after real PES (lower array) according to frequency bands alpha, beta, low gamma; N = 10. Negative values denote ERD. L = left; R = right.

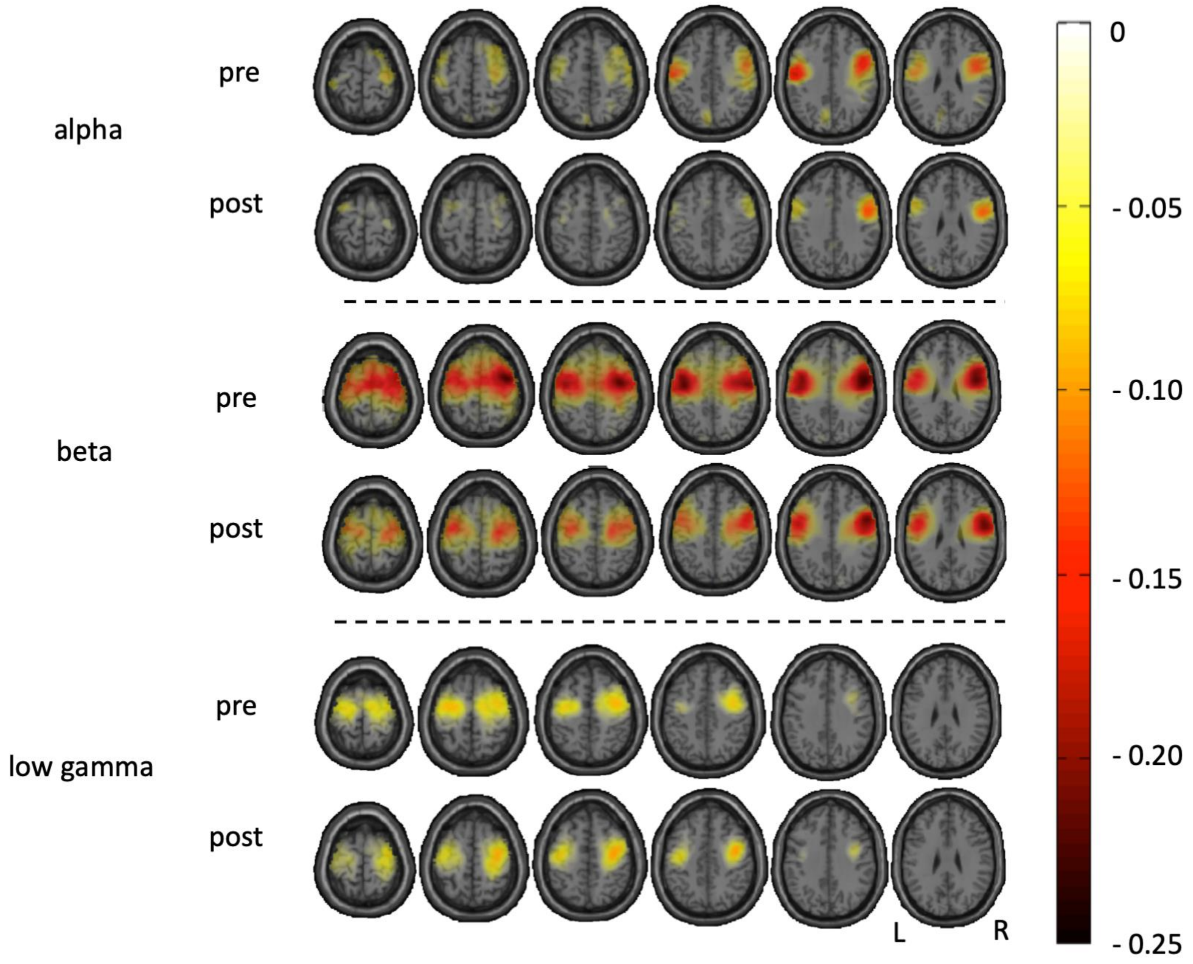

<u>Supplementary figure 3:</u> Swallowing-related group mean ERD before (upper array) and after sham-PES (lower array) according to frequency bands alpha, beta, low gamma; N = 10. Negative values denote ERD. L = left; R = right.

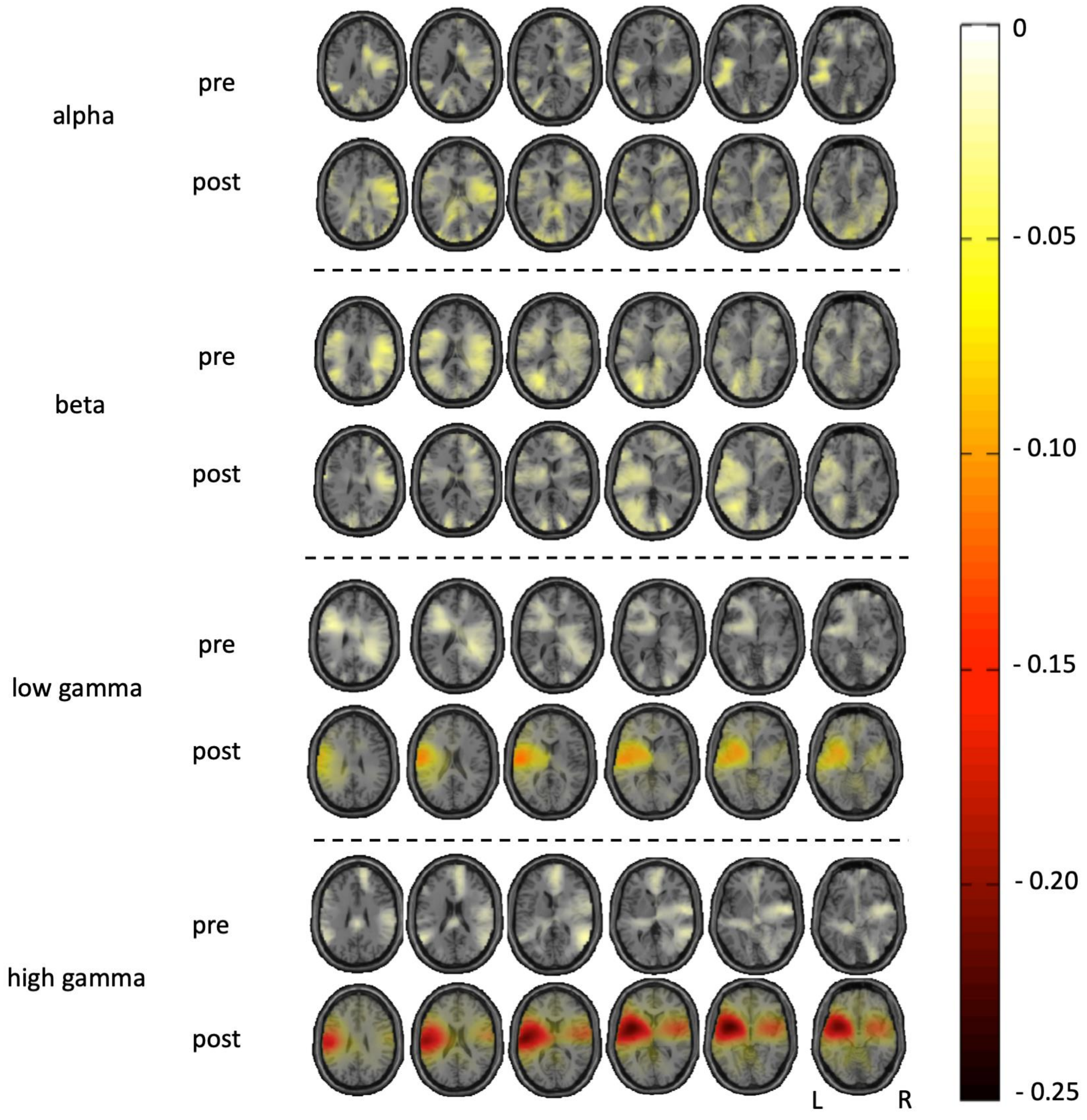

Supplementary figure 4:

Group mean ERD related to pharyngeal air-puff stimulation before (upper array) and after real PES (lower array) according to frequency bands alpha, beta, low gamma, high gamma; N = 10. Negative values denote ERD. L = left; R = right.

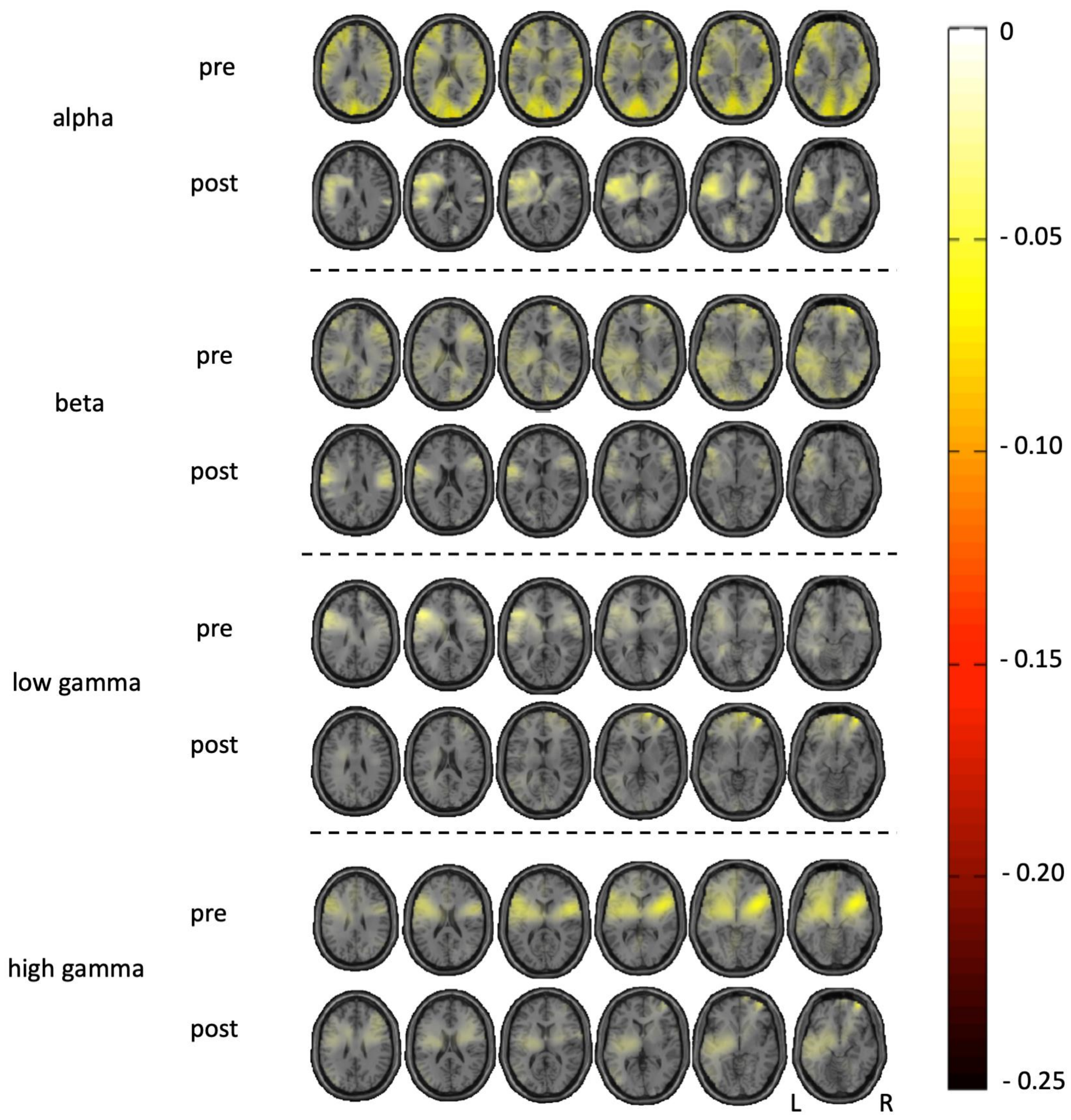

Supplementary figure 5:

Group mean ERD related to pharyngeal air-puff stimulation before (upper array) and after sham-PES (lower array) according to frequency bands alpha, beta, low gamma, high gamma; N = 10. Negative values denote ERD. L = left; R = right
